# Development of the short Creative Expression Interest Scale based on item response theory

**DOI:** 10.3389/fpsyg.2022.955176

**Published:** 2022-09-22

**Authors:** Peng Juan Zhao, Xu Liang Gao, Nan Zhao, Zhao Sheng Luo

**Affiliations:** ^1^School of Psychology, Guizhou Normal University, Guiyang, China; ^2^School of Education, Jiangxi Normal University, Nanchang, China; ^3^School of Psychology, Jiangxi Normal University, Nanchang, China

**Keywords:** short scale, Creative Expression Interest Scale, item response theory, personality, self-efficiency

## Abstract

This study develops a short Creative Expression Interest Scale (CEIS) among Chinese freshmen based on the perspective of item response theory (IRT). Nine hundred fifty-nine valid Chinese freshmen participated in the Creative Expression Interest survey. Researchers applied the initial data for unidimensionality, item fit, discrimination parameter, and differential item functioning to obtain a short CEIS. The results show that the Short CEIS meets the psychometric requirements of the IRT. Pearson correlation coefficient of theta between the short and long CEIS is 0.922. The marginal reliability of the short CEIS is 0.799. These indicate that the short CEIS developed in this study among Chinese freshmen, meets the psychometric requirements. Although the Short CEIS can eliminate redundant, uninformative items, save time, and improve the quality of data collection. However, the validity of this short scale needs further validation.

## Introduction

Interest is essential in the vocational, organizational, and educational psychology fields. Vocational interest is an individual's preference and good at specific activities, which can stimulate an individual's goal-directed behavior and have greater predictive power for his or her engagement in a particular job (Su et al., 2019a). The current research on vocational interest is mainly based on Holland Vocational Interest Scale to explore the relationship between interest, education, and occupation (Su, 2020). In the occupational field, the type of interest is essential to the characteristics of the unique working environment (Hoff et al., 2020a) and life purpose (Stoll et al., 2021). Interest is also closely related to job performance (Van Iddekinge et al., 2011; Nye et al., 2017) and positively predicts job satisfaction (Hoff et al., 2020b) and career success (James and Su, 2014). In the field of education, interest is an emotional state which is an intrinsic motivation that drives learning and can predict academic achievement (Leung et al., 2014; Nye et al., 2021). Meta-analysis finds that occupational interest is relatively stable (Low et al., 2005; Hoff et al., 2018, 2021; Stoll et al., 2021). Therefore, it is vital for freshmen to objectively and comprehensively evaluate their vocational interests. CEIS is essential for students majoring in art or interested in this field. When a student takes part in the Creative Expression interest test, he or she can make a simple assessment of his/her interest and receive career guidance before applying for a job. At the same time, career interest tests help art students to better understand their majors, improve their professional identity, plan their careers well in advance, and increase employment success.

Although vocational interest has been generally recognized, there is no uniform conclusion on the division of specific dimensions (Gati, 1991). The vocational interest structure mainly focuses on dimensions four, six, seven, and eight. Turnstone developed a four-dimensional vocational interest scale based on a version of the Strong scale (Thurstone, 1931). The four-dimensional vocational interest concludes with Science, Language, People, and Business aspects. The four-dimensional vocational interest concludes with Science, Language, People, and Business aspects. The Language dimension encompasses advertising, art, and news. Guilford used analytic and clustering methods to develop a version with seven dimensions-scientific, aesthetic expression, social welfare, business, clerical, mechanical, and outdoor work. This version is the aesthetic expression that includes music, literature, drama, and artistic performance (Guilford et al., 1954). Jackson (Jackson, 1977) thought vocational interest should have logical, inquiring, expressive communication, and help conventional practical enterprising. Holland (Holland, 1959) proposed a new vocational interest scale which is popular at present. This scale has six dimensions-RIASEC (Realistic, Investigative, Artistic, Social, Enterprising, and Conventional). Based on the previous studies and the 2018 Standard Occupational Classification (SOC) version, Su developed an 8-dimensions model (SETPOINT: Health Science, Creative Expression, Technology, People, Organization, Influence, Nature, and Things). Creative expression has Media, Applied Arts & Design, Music, Visual Arts, Performing Arts, Creative Writing, and Culinary Art (Su et al., 2019b).

With the development of the times, the structure of vocational interest is constantly changing. So far, no vocational interest scale fits all venues (Liu and Rounds, 2003). However, based on existing models, it is easily found that art interest has always existed. Only the way of Expression and content has changed. The search for a dimensional structure of interests began with Thurstone's factor analysis. In this version, the language dimension has advertising, art, law, ministry, and journalism. One might guess that these professions are characterized more or less by an interest in talk (Thurstone, 1931). Later, studies began to use a variety of interest inventories. It is crucial to note Guilford's content-specific essential interest scales. This version's aesthetic Expression includes music, literature, drama, and artistic performance (Guilford et al., 1954). Additionally, in the late 1970s, Rounds et al. used many interest scales and included female and male participants. They labeled a dimension aesthetics such as sculptor, writing a one-act play, music composer, scenario writer, and illustrator (Rounds and Dawis, 1979). Holland defined this aspect as artistic in his six-dimension model (Holland, 1959). In one eight-dimension model, Creative Expression has Media, Applied Arts & Design, Music, Visual Arts, Performing Arts, Creative Writing, and Culinary Art (Su et al., 2019b). These expressions reflect the same underlying interest despite individuals expressing their creativity differently. Therefore, this study uses CEIS, a vocational interest scale developed by (Su et al., 2019b). Creative expression captures a general interest in activities involving expressing imaginative and creative ideas in various forms for art or practical considerations (Su et al., 2019b).

Scholars are always concerned about the quality of questionnaire responses (Kraut et al., 1975; Herzog and Bachman, 1981; Zhao and Kang, 1988; Burisch, 1997; Bowling et al., 2016). Long scales increase the cognitive load of the respondent. Subjects are easily fatigued. When a person is in this state, the quality of his or her answer will decrease (Meade and Craig, 2012; Gibson and Bowling, 2020; Zhong et al., 2021). There are many advantages of short scales, such as saving time, reducing the minimum number of subjects required, and improving the quality of responses to the data (Russell et al., 1989; Robins et al., 2001; Abdel-Khalek, 2006; Postmes et al., 2013).

When conducting studies with large samples, researchers recommend using single-item scales due to time constraints (Zhang and Wei, 2019; Goldammer et al., 2020). Career interest assessment is the basis for career planning. CEIS contains seven dimensions (Su et al., 2019b). If one item is extracted from a dimension, the short version of CEIS maybe contained seven items. Above all, we have a hypothesis: short CEIS has seven items. The seventh dimension is related to food processing and catering. In China, the traditional view is that food processing and catering belong to the service industry. Few people would associate these with art. Thus, there may be six items in the short CEIS.

The concepts and procedures of item response theory (IRT) have much broader applicability for psychological measurement (Steinberg and Thissen, 1996; Ark, 2007). This theory is through the item response curve synthesis of all item analysis data so that we can comprehensively and intuitively see the item difficulty, discrimination, and other characteristics. The most significant advantage of IRT is the invariance of question parameters. That is to say, the estimation of question parameters is independent of the subject group. This advantage helps to revise or develop scale. However, based on IRT, estimating the reliability and validity of the vocational interest scale is very rare (Tay et al., 2009). Therefore, the current research aims to use IRT and develop a short CEIS among Chinese freshmen.

General procedures for scale revision are as follows: first, evaluate assumptions of the IRT Model for unidimensionality, local independence, and monotonicity; second, Fit the IRT model to data, such as examine model fit, evaluate item properties, evaluate scale properties; third, evaluate differential item functioning (DIF) between gender groups (Reeve et al., 2007).

## Samples

The researchers used a convenience sampling method to recruit subjects for this study. One thousand and seventy-two Chinese freshmen took part in the questionnaire survey. They came from Henan, Jiangxi, Guizhou, and Guangdong provinces of China. The study involved human participants and was reviewed and approved by the morality and ethics committee of the School of Psychology, Guizhou Normal University. The participants provided oral informed consent to participate in this study. Before conducting the questionnaire, each participant was informed of the individual privacy protection principle, and then they volunteered to participate in the survey. The survey included the basic demographic information (gender), the CEIS, and questions used as the exclusion criteria. In order to obtain accurate and effective response data, we screened the original questionnaires in advance.

The researchers distributed 1,072 questionnaires, and 1,000 completed questionnaires were collected, with a recovery rate of 93.28%. The final valid data for this study was 959, suggesting that the valid rate was 95.9%. If a participant's response at least met one aspect standard in the following, his or her response was regarded as invalid data: The two questions that measure attitudes do not meet the requirements (one was ‘this question is no response, please skip it,’ the other question was ‘Please choose choice A for this question’); there were clear answering rules, not thoughtfully answering; there were more than or equal two missing items. At last, there were 959 valid data. In this study, the researchers adopted a convenient snowball method; simultaneously, participants followed the principle of voluntary. As a result, the ratio of male to female students is unbalanced. Among participants, 282 were male (29.406%), and 677 were female (70.594%). One hundred three majored in engineering, 297 majored in education, 149 majored in science, 125 majored in literature, 139 majored in art, and 139 majored in others.

## Measure

This study adopted the CEIS developed by (Su et al., 2019b). This part has 28 items on a five-point Likert scale (1 = Dislike a great deal, 5 = Like a great deal). The original long scale has media, applied arts & design, music, visual arts, performing arts, creative writing, and culinary art 7 dimensions. All the items belong to Creative Expression Interest. That is to say. This scale is a congeneric model (Osburn, 2000). Creative Expression Interest is one dimensional of vocational interest, so the higher the score is, the higher the level of Creative Expression Interest.

## Procedure

Before an IRT model can be fit to data, three assumptions must be met. First, Unidimensionality, local independence, and monotonicity are the essential condition for IRT (Drasgow and Parsons, 1983; Embretson and Reise, 2000; Reeve et al., 2007; Acevedo-Mesa et al., 2021). Through the above steps of the IRT analysis, items met all the criteria to assess the person's ability parameter, then evaluate differential item functioning, calculate the correlation of personal parameters (theta) between the original (long) scale and the revised (short) scale.

## Unidimensionality

In IRT, unidimensionality implies that one major ability or trait should explain or account for the test performance of examines (Hambleton and Swaminathan, 2014). The criteria for the data might be reasonably well fitted by a unidimensional model through exploratory factor analysis (EFA). The ratio of the first eigenvalue to the second eigenvalue was above 3 (Hattie, 1985). The percentage of variance interpreted by the first factor was more than 20% of the total variance (Reckase, 1979). Only two criteria were satisfied simultaneously; items might fit the unidimensionality model well (Reckase, 1979). The data on Creative Expression Interest might fit the unidimensionality model well. A bifactor model was used for the confirmatory factor analysis (CFA) (Li et al., 2015). Then researchers, *via* the bifactor function in the psych package of software R4.1.1, conducted a confirmatory factor analysis to test the unidimensionality in the way of high-order bifactor analysis (Gibbons and Hedeker, 1992; Reise et al., 2013; Sunderland et al., 2020). Model fit statistics were used to evaluate model fit and a select optimal number of specific factors and omega.h (ω_*h*_) coefficient. The ω_*h*_ coefficient can be interpreted as the variance in unit-weighted total scores attributable to a single general factor, treating variability in scores due to group factors as measurement error (Sunderland et al., 2020). If ω_*h*_ > 0.70, we can assume that the overall scores influence a single source primarily and provide support for essential unidimensionality (Sunderland et al., 2020).

### Local independence

Local independence assumes that once the dominant factor influencing a person's response to an item is controlled, there should be no significant association among item responses (Ark, 2007). The existence of local independence that influences IRT parameter estimates poses a problem for scale construction. Uncontrolled local independence (LD) among items in an IRT assessment could result in a score different from the measured artistic expression interest construct. High residual correlations (greater than 0.2) will be flagged and considered as possible LD. If two items' LD coefficients were more significant than 0.2, it is better to consider deleting this item (Chen and Thissen, 1997). This function is residuals in the R4.1.1 Lavaan package.

### Monotonicity

The assumption of monotonicity means that the probability of endorsing or selecting an item response indicative of a more vital interest status should increase as the underlying level of preference interest. Therefore, monotonicity is an essential requirement for IRT models for items with ordered response categories. In this process, we computed monotonicity *via* the R4.1.1 Mokken package. In addition, Loevinger was the first to develop an explicit theory of homogeneous tests based on “cumulative” or monotone items (Loevinger, 1948). Homogeneity (H) is one coefficient of monotonicity (Mokken, 1971). In this study, we choose the maximum test score (Hi) to be more than 0.3 (Ark, 2007).

### Fit item response theory (IRT) model to data

Estimate IRT model parameters; estimate IRT model relative fitting index; examine model fit; evaluate item properties, category response curves, and item information curves; evaluate scale properties, information function (Reeve et al., 2007).

### Estimate IRT model relative fitting index

To assess the degree of fitness for the whole items, the graded response model (GRM) (Samejima, 1969), generalized partial credit model (GPCM) (Muraki, 1992), and Rating Scale Model (RSM) (Andrich, 1978) dealing with polychromous-scored items were used in this study. Given the two test-level model-fit indices: Akaike's information criterion (*AIC*) (Akaike, 1974), and Bayesian information criterion (*BIC*) (Schwarz, 1978), the model representing the indices with relatively more minor values were taken as the optimal model for the subsequent analysis. Smaller values of these test-fit indices indicate better model fit (Tan et al., 2018). Therefore, we chose the most miniature model to compare the three model parameters (Reeve et al., 2007). In this process, we use the R4.1.1 Mirt package (Chalmers, 2012).

### Item selection

After fitting the selected model, we considered the item content and the resulting psychometric information, such as item response and item information curves. These curves are used to evaluate item quality together with other criteria to identify items for removal from the original version or not.

### Item fit

After comparing the relative goodness-of-fit indices of the models, the optimal model was selected, and the overall degree of data fitting was suggested. However, this did not indicate that each item could fit the optimal model well. Accordingly, the degree of item-fit further should be explored. Compare observed and expected response frequencies or examine fit indices, usually using S-χ^2^ (Reeve, 2003), which quantifies and compares the differences between observed and expected frequencies under the IRT model. The S-χ^2^ was adopted to show the degree of the item-fit index (Orlando, 2000; Maria and David, 2003). Items with a *p*-value of S-χ^2^ <0.001 were considered poor item-fit (Reeve et al., 2007; Flens et al., 2017). It would be considered as a low item-fit one and then removed.

## Discrimination

Discrimination represents an item's ability to discriminate between individuals high and low on the latent trait (Li et al., 2015). An item with high discrimination implies that this item is preferable to distinguish whether individuals exhibit signs of preference. Therefore, a high discrimination parameter of one item suggests that this item is of high quality and helps in obtaining a more precise estimation of a latent population trait. We used the pars function by the R stats package to estimate item parameters *via* the optimal model based on a test-level model-fit check and chose items with discrimination of more than 0.8 (*a* > 0.8) (Tan et al., 2018).

### Evaluate differential item functioning (DIF)

Considering the importance of the equivalence test in practical tests, the authors use DIF analysis to evaluate the systematic error caused by group bias. The DIF analysis can identify systematic errors attributed to different groups. That is, we conducted the DIF analysis to determine whether an individual's response to an item was a function of the underlying latent trait but of gender as well. If an item had DIF, the probability of selecting the same item type might differ for groups at the comparable latent trait level. The current study investigated DIF for gender (male/female). There were lots of methods, such as logistic regression (Crane et al., 2006), Likelihood-ratio (Orlando Edelen et al., 2006), and Wald tests (Millsap and Everson, 1993) to conduct the DIF analysis. Wald test compares separately estimated parameters across the male and female groups to examine whether they are statistically similar. These statistics could be used in tests of polytomous models. These models require more parameters than in the dichotomous case. The *p* < 0.001 revealed a functional difference in the item, which should be removed (Reeve et al., 2007). In order to reserve more items, we chose the *p*-value threshold of 0.001. In the process, researchers used the method of Wald to evaluate differential item functioning by the R Mirt package (Chalmers, 2012).

## Pearson correlation between short and original CEIS

There are two CEIS. One version is the original long CEIS, and the other is a revised short CEIS. In this section, researchers computed every sample's theta, both long and short versions. First, expect a posteriori (EAP) was used to calculate the sample's theta. Second, researchers computed the theta coefficient of Pearson's correlation between the two versions *via* the Cor function in the R4.1.1 Psych package. This study then calculated the Pearson correlation between the CTT scores of short CEIS and each item that came from the short version.

## Evaluate assumptions of the item response theory (IRT) model

Before applying IRT models, it is essential to evaluate the core assumptions of the model, for example, unidimensionality, monotonicity, and local independence. The ratio of the first and second components was 4.114, exceeding the criteria of 3 (Hattie, 1985). The first factor interpreted about 38.3% of all the variance, which was more than the criteria of 20% (Reckase, 1979). At the same time, researchers used high-order Bifactor analysis and got a coefficient of approximately 0.788, which was more than 0.70 (Sunderland et al., 2020). The above results indicated that the remained items could fit the unidimensional model well. Hi, values of all items were more significant than 0.3 (Ark, 2007), which met the monotonicity condition. The probability of agreeing or selecting a more substantial interest is increased with the level of interest (see Table 1). Pairs of items were reviewed for possible local dependence (LD) when residual correlations were more remarkable than 0.2 (Reeve et al., 2007). From Table 2, according to the Criteria of LD < 0.2, only seven items which are item3, item5, item9, item13, item17, item21, and item25, met the requirements (see Table 1).

**Table 1:** Local independence.

**Item**	**Item1**	**Item2**	**Item3**	**Item4**	**Item5**	**Item6**	**Item7**	**Item8**	**Item9**	**Item10**	**Item11**	**Item12**	**Item13**	**Item14**	**Item15**	**Item16**	**Item17**	**Item18**	**Item19**	**Item20**	**Item21**	**Item22**	**Item23**	**Item24**	**Item25**	**Item26**	**Item27**	**Item28**
Item1	NA	0.246	0.197	0.166	−0.129	0.124	−0.157	−0.135	−0.106	−0.141	−0.133	−0.163	−0.121	−0.123	−0.116	−0.130	0.106	0.124	0.124	0.114	−0.118	−0.106	−0.154	−0.139	−0.106	−0.100	−0.093	−0.112
Item2	NA	NA	0.212	0.222	−0.151	0.139	−0.178	−0.156	−0.112	−0.126	−0.137	−0.157	−0.147	−0.124	−0.143	−0.139	−0.128	−0.111	−0.126	−0.125	0.141	0.119	0.111	0.085	−0.089	−0.109	−0.108	−0.113
Item3	NA	NA	NA	0.270	−0.156	−0.153	−0.126	−0.16	−0.105	−0.166	−0.132	−0.172	−0.164	−0.168	−0.136	−0.147	0.140	0.126	0.127	−0.115	−0.125	−0.152	−0.133	−0.149	−0.113	−0.127	−0.137	−0.145
Item4	NA	NA	NA	NA	−0.160	0.178	−0.154	−0.175	−0.140	−0.183	−0.151	−0.186	−0.138	−0.122	−0.122	−0.133	−0.132	−0.125	−0.118	−0.119	0.127	−0.146	−0.154	−0.133	−0.134	−0.147	−0.134	−0.143
Item5	NA	NA	NA	NA	NA	0.232	0.220	0.250	−0.156	−0.203	−0.173	−0.182	0.139	0.115	0.121	0.142	−0.175	−0.147	−0.141	−0.131	−0.151	−0.155	−0.145	−0.135	0.112	0.121	0.118	0.139
Item6	NA	NA	NA	NA	NA	NA	0.324	0.254	−0.129	−0.200	−0.157	−0.209	−0.125	−0.106	−0.099	−0.129	−0.139	−0.131	−0.148	−0.127	−0.138	−0.134	−0.141	−0.146	−0.129	−0.148	−0.138	−0.135
Item7	NA	NA	NA	NA	NA	NA	NA	0.285	−0.135	−0.185	−0.158	−0.182	−0.144	−0.130	−0.112	−0.139	−0.166	−0.153	−0.138	−0.137	−0.148	−0.166	−0.159	−0.174	−0.128	−0.117	−0.129	−0.137
Item8	NA	NA	NA	NA	NA	NA	NA	NA	−0.147	−0.216	−0.173	−0.225	0.143	0.133	0.126	0.163	−0.174	−0.162	−0.178	−0.140	−0.160	−0.161	−0.153	−0.161	−0.133	−0.123	−0.122	−0.136
Item9	NA	NA	NA	NA	NA	NA	NA	NA	NA	0.285	0.301	0.204	−0.165	−0.156	−0.150	−0.142	0.145	0.126	−0.129	−0.106	−0.119	−0.144	−0.139	−0.147	−0.122	−0.126	−0.130	−0.151
Item10	NA	NA	NA	NA	NA	NA	NA	NA	NA	NA	0.340	0.264	−0.190	−0.167	−0.175	−0.133	0.149	0.143	0.146	−0.140	−0.141	−0.149	−0.152	−0.138	−0.131	−0.150	−0.137	−0.160
Item11	NA	NA	NA	NA	NA	NA	NA	NA	NA	NA	NA	0.319	−0.193	−0.167	−0.186	−0.149	0.206	0.154	0.145	0.143	−0.143	−0.166	−0.144	−0.114	−0.161	−0.142	−0.158	−0.150
Item12	NA	NA	NA	NA	NA	NA	NA	NA	NA	NA	NA	NA	−0.193	−0.173	−0.195	−0.175	0.179	0.151	0.136	0.125	−0.158	−0.185	−0.168	−0.141	−0.149	−0.150	−0.148	−0.145
Item13	NA	NA	NA	NA	NA	NA	NA	NA	NA	NA	NA	NA	NA	0.463	0.336	0.233	−0.150	−0.157	−0.167	−0.165	−0.160	−0.173	−0.159	−0.172	−0.118	−0.137	−0.138	−0.140
Item14	NA	NA	NA	NA	NA	NA	NA	NA	NA	NA	NA	NA	NA	NA	0.404	0.245	−0.161	−0.164	−0.179	−0.168	−0.143	−0.180	0.149	−0.153	−0.131	−0.123	−0.131	−0.151
Item15	NA	NA	NA	NA	NA	NA	NA	NA	NA	NA	NA	NA	NA	NA	NA	0.262	−0.175	−0.152	−0.173	−0.155	−0.155	−0.169	−0.124	−0.127	−0.125	−0.115	−0.119	−0.130
Item16	NA	NA	NA	NA	NA	NA	NA	NA	NA	NA	NA	NA	NA	NA	NA	NA	−0.151	−0.139	−0.161	−0.148	−0.145	−0.148	−0.145	−0.161	−0.128	−0.152	−0.138	−0.128
Item17	NA	NA	NA	NA	NA	NA	NA	NA	NA	NA	NA	NA	NA	NA	NA	NA	NA	0.262	0.230	0.208	−0.145	−0.177	−0.160	−0.157	−0.141	−0.140	−0.154	−0.159
Item18	NA	NA	NA	NA	NA	NA	NA	NA	NA	NA	NA	NA	NA	NA	NA	NA	NA	NA	0.436	0.309	−0.132	−0.150	−0.142	−0.133	−0.115	−0.114	−0.123	0.135
Item19	NA	NA	NA	NA	NA	NA	NA	NA	NA	NA	NA	NA	NA	NA	NA	NA	NA	NA	NA	0.313	−0.160	−0.171	−0.171	−0.159	−0.117	−0.127	−0.128	−0.140
Item20	NA	NA	NA	NA	NA	NA	NA	NA	NA	NA	NA	NA	NA	NA	NA	NA	NA	NA	NA	NA	−0.171	−0.185	−0.161	−0.149	−0.125	−0.142	−0.121	−0.136
Item21	NA	NA	NA	NA	NA	NA	NA	NA	NA	NA	NA	NA	NA	NA	NA	NA	NA	NA	NA	NA	NA	0.440	0.240	0.239	−0.119	−0.127	−0.104	−0.122
Item22	NA	NA	NA	NA	NA	NA	NA	NA	NA	NA	NA	NA	NA	NA	NA	NA	NA	NA	NA	NA	NA	NA	0.277	0.272	−0.147	−0.143	−0.125	−0.141
Item23	NA	NA	NA	NA	NA	NA	NA	NA	NA	NA	NA	NA	NA	NA	NA	NA	NA	NA	NA	NA	NA	NA	NA	0.391	−0.128	−0.121	−0.110	−0.130
Item24	NA	NA	NA	NA	NA	NA	NA	NA	NA	NA	NA	NA	NA	NA	NA	NA	NA	NA	NA	NA	NA	NA	NA	NA	−0.140	−0.151	−0.131	−0.139
Item25	NA	NA	NA	NA	NA	NA	NA	NA	NA	NA	NA	NA	NA	NA	NA	NA	NA	NA	NA	NA	NA	NA	NA	NA	NA	0.435	0.421	0.349
Item26	NA	NA	NA	NA	NA	NA	NA	NA	NA	NA	NA	NA	NA	NA	NA	NA	NA	NA	NA	NA	NA	NA	NA	NA	NA	NA	0.524	0.379
Item27	NA	NA	NA	NA	NA	NA	NA	NA	NA	NA	NA	NA	NA	NA	NA	NA	NA	NA	NA	NA	NA	NA	NA	NA	NA	NA	NA	0.413
Item28	NA	NA	NA	NA	NA	NA	NA	NA	NA	NA	NA	NA	NA	NA	NA	NA	NA	NA	NA	NA	NA	NA	NA	NA	NA	NA	NA	NA

**Table 2:** Estimate item response theory (IRT) model relative fit index.

**Model**	**AIC**	**BIC**	**Loglik**
Generalized Partial Credit Model (GPCM)	71256.109	71937.334	−35488.050
Graded Response Model (GRM)	70693.579	71374.804	−35206.790
Rating Scale Model(RSM)	71802.870	71958.579	−35869.440

AIC, Akaike's information criterion. BIC, Bayesian information criterion. Loglik, Log- likelihood.

## Test fit and IRT model selection

Test fit statistics of the GRM, the GPCM, and the RSM were documented in Table 2. For the GRM, AIC = 70693.579, and BIC = 71374.804, −2ll = 70413.58 (Log-likelihood = −35206.79). All relative fit indices of the GRM were less than those of the other two IRT models, which suggested that the GRM fitted the data better than the others. Therefore, this study chose the GRM model in the following parts (Reeve et al., 2007).

## Item model-fit, discrimination, and different item functioning

**Figure 1:**
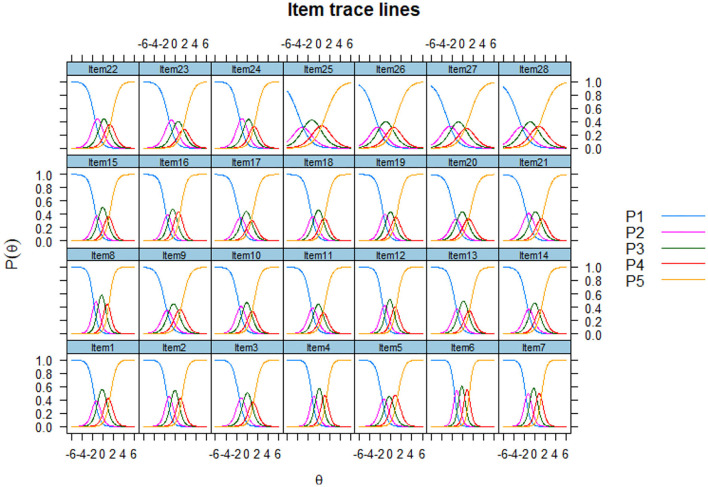
Item response curves.

**Figure 2:**
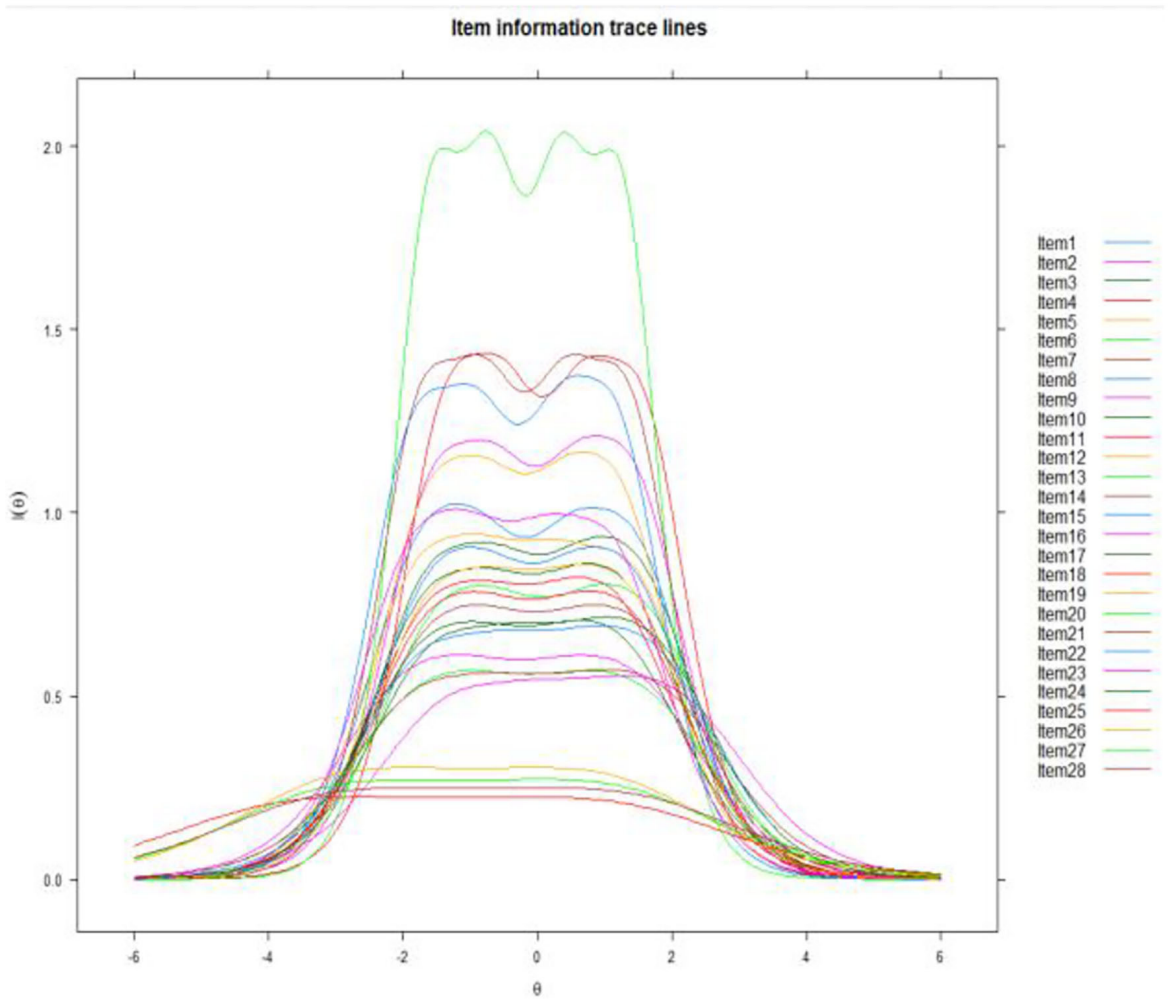
Item information curves.

Items with *p* values of *P.s-x2* less than 0.001 were poor item-fit and should be eliminated (see Table 3). The *P.s-x2* values of all items were more remarkable than 0.001, reaching the index of fitting degree test (Reeve et al., 2007). Therefore, there was no item removed. The discrimination coefficient of all items is more significant than 0.80, whose discrimination values are less than 0.8 and should be excluded from the items (Cho et al., 2015). A high discrimination parameter of one item suggests that this item is of high quality and helps obtain a more precise estimation of a latent population trait. Difficulty coefficient b is another index. From Figure 1, curve 1 decreases monotonically, curves 2, 3, and 4 firstly increase and then decrease, curve 5 monotonically increases, and curve 1 generally has a significant slope. The specific ability of 28 items is better and conforms to the item characteristic curves of the ideal state. The values of the b parameter should be in the range of (– 3, 3) (Steinberg and Thissen, 1996). The first 24 items' b parameter is in this range, while the values of the b parameter of Item25, Item26, Item27, and Item28 are beyond this scope. Samples of the original scale came from the Purdue University and the U.S. workforce (Su et al., 2019b), where a cultural concept is different from China. Few people in China were associated with food processing and catering with the artist. Four item information curves are relatively flat (Figure 2). These are consistent with the mean item information (mean-IIF) in Table 3. Item25, Item26, Item27, and Item28 can't distinguish different subjects' abilities well (Reeve et al., 2007). From this criterion, these four items should be deleted. According to Table 4, it is easily found that if the *p* value is taken as significance at 0.001 levels, four items should be deleted from the DIF test. They are Item4, Item13, Item14, and Item15. Item13, Item14, and Item15 are three items all belong to visual arts. If we removed all the four items, CEIS would lack visual arts (such as, Sketch a picture). According to the Criteria of LD and other indexes, Item13 fits the requirements. Considering that Item13 meets the criteria in all other criteria and ensures structural integrity; finally, Item13 is still in the expressive art interest scale.

**Table 3:** Graded response model (GRM)'s item parameters.

**Item**	**Content**	**Hi**	***a***	***b*_1_**	***b*_2_**	***b*_3_**	***b*_4_**	**Mean-IIF**	**p.S_X2**
Item1	Direct a TV show	0.424	1.853	−1.754	−0.869	0.492	1.466	0.951	0.412
Item2	Write a movie screenplay	0.443	2.023	−1.578	−0.622	0.584	1.472	1.127	1.000
Item3	Host a radio program	0.407	1.762	−1.629	−0.586	0.679	1.553	0.879	0.540
Item4	Develop a podcast series	0.453	2.219	−1.388	−0.508	0.663	1.569	1.323	0.630
Item5	Create a piece of artistic and functional furniture	0.414	1.772	−1.816	−0.827	0.278	1.435	0.895	0.082
Item6	Create the set for a movie or stage play	0.484	2.699	−1.601	−0.696	0.341	1.255	1.869	0.923
Item7	Design the layout and lighting of an exhibition	0.453	2.236	−1.741	−0.775	0.397	1.367	1.341	0.016
Item8	Design unique packaging for a product	0.445	2.166	−1.857	−0.891	0.333	1.211	1.264	0.214
Item9	Play a musical instrument	0.377	1.419	−1.984	−0.948	0.415	1.491	0.591	0.625
Item10	Compose an original piece of music	0.403	1.683	−1.682	−0.649	0.551	1.388	0.815	0.162
Item11	Play in a band	0.408	1.642	−1.682	−0.695	0.468	1.263	0.779	0.368
Item12	Arrange background music for a show	0.440	1.975	−1.65	−0.737	0.412	1.265	1.087	0.249
Item13	Sketch a picture	0.401	1.631	−1.522	−0.558	0.764	1.647	0.761	0.180
Item14	Paint a landscape	0.396	1.572	−1.631	−0.653	0.622	1.593	0.717	0.393
Item15	Draw illustrations for a book	0.414	1.735	−1.618	−0.713	0.568	1.446	0.854	0.831
Item16	Create a unique piece of artwork	0.433	1.833	−1.887	−0.986	0.151	1.166	0.947	0.473
Item17	Perform on stage for a group of people	0.385	1.52	−1.745	−0.772	0.482	1.314	0.673	0.012
Item18	Act in a play	0.406	1.61	−1.661	−0.713	0.544	1.431	0.749	0.175
Item19	Act out an emotional movie scene	0.412	1.681	−1.614	−0.605	0.515	1.404	0.817	0.164
Item20	Perform comedy to entertain an audience	0.366	1.365	−1.799	−0.8	0.573	1.586	0.551	0.619
Item21	Write a novel	0.369	1.369	−1.819	−0.531	0.843	1.859	0.553	0.669
Item22	Write short stories	0.389	1.507	−1.735	−0.471	0.769	1.762	0.664	0.484
Item23	Compose a poem	0.368	1.342	−1.367	−0.024	1.244	2.125	0.528	0.275
Item24	Study creative writing	0.397	1.53	−1.58	−0.338	0.881	1.773	0.681	0.719
Item25	Select ingredients to prepare food	0.309	0.857	−3.934	−2.414	−0.308	1.327	0.219	0.494
Item26	Create the recipe for a new dish	0.337	0.995	−3.126	−1.808	−0.100	1.207	0.296	0.978
Item27	Create a new cooking technique to enhance flavor	0.321	0.940	−3.211	−1.746	0.037	1.350	0.266	0.931
Item28	Learn about required temperature and time for baking pastries	0.303	0.901	−3.278	−1.796	0.061	1.572	0.246	0.517

*N* = 959. GRM, Graded Response Model. *Hi*, Homogeneity (H), *a* coefficient of scalability (monotonicity) in terms of manifest parameters. *a*, item discrimination parameter. *b*_1_- *b*_4_, item difficulty. *b*_1_, difficulty parameter between response categories 1 and 2. *b*_2_, difficulty parameter between response categories 2 and 3. *b*_3_, difficulty parameter between response categories 3 and 4. *b*_4_, difficulty parameter between response categories 4 and 5. mean-IIF, mean item information. *p.S_X*^2^, Item fitting index.

**Table 4:** Gender differential item functioning.

**Item**	**Converged**	**AIC**	**BIC**	**χ^2^**	***df***	***p***	***adj*- *p***
Item1	TRUE	9.630	14.699	19.630	5	0.001	0.004
Item2	TRUE	8.867	15.462	18.867	5	0.002	0.005
Item3	TRUE	13.347	10.983	23.347	5	0.000	0.002
Item4	TRUE	35.825	11.495	45.825	5	0.000	0.000
Item5	TRUE	1.949	22.380	11.949	5	0.035	0.062
Item6	TRUE	10.446	13.883	20.446	5	0.001	0.004
Item7	TRUE	11.755	12.574	21.755	5	0.001	0.003
Item8	TRUE	4.861	19.468	14.861	5	0.011	0.022
Item9	TRUE	5.794	18.535	15.794	5	0.007	0.016
Item10	TRUE	9.615	14.715	19.615	5	0.001	0.004
Item11	TRUE	0.937	25.267	9.063	5	0.107	0.142
Item12	TRUE	1.824	26.154	8.176	5	0.147	0.187
Item13	TRUE	20.507	3.822	30.507	5	0.000	0.000
Item14	TRUE	16.587	7.742	26.587	5	0.000	0.000
Item15	TRUE	31.383	7.053	41.383	5	0.000	0.000
Item16	TRUE	9.906	14.424	19.906	5	0.001	0.004
Item17	TRUE	3.328	27.657	6.672	5	0.246	0.265
Item18	TRUE	0.267	24.597	9.733	5	0.083	0.131
Item19	TRUE	2.124	26.454	7.876	5	0.163	0.199
Item20	TRUE	4.422	28.751	5.578	5	0.349	0.362
Item21	TRUE	2.207	22.122	12.207	5	0.032	0.060
Item22	TRUE	0.455	24.785	9.545	5	0.089	0.131
Item23	TRUE	6.752	17.577	16.752	5	0.005	0.012
Item24	TRUE	2.966	27.295	7.034	5	0.218	0.254
Item25	TRUE	3.149	27.479	6.851	5	0.232	0.260
Item26	TRUE	0.605	24.935	9.395	5	0.094	0.132
Item27	TRUE	7.346	31.675	2.654	5	0.753	0.753
Item28	TRUE	0.430	24.760	9.570	5	0.088	0.131

AIC, Akaike's information criterion. BIC, Bayesian information criterion.

**Figure 3:**
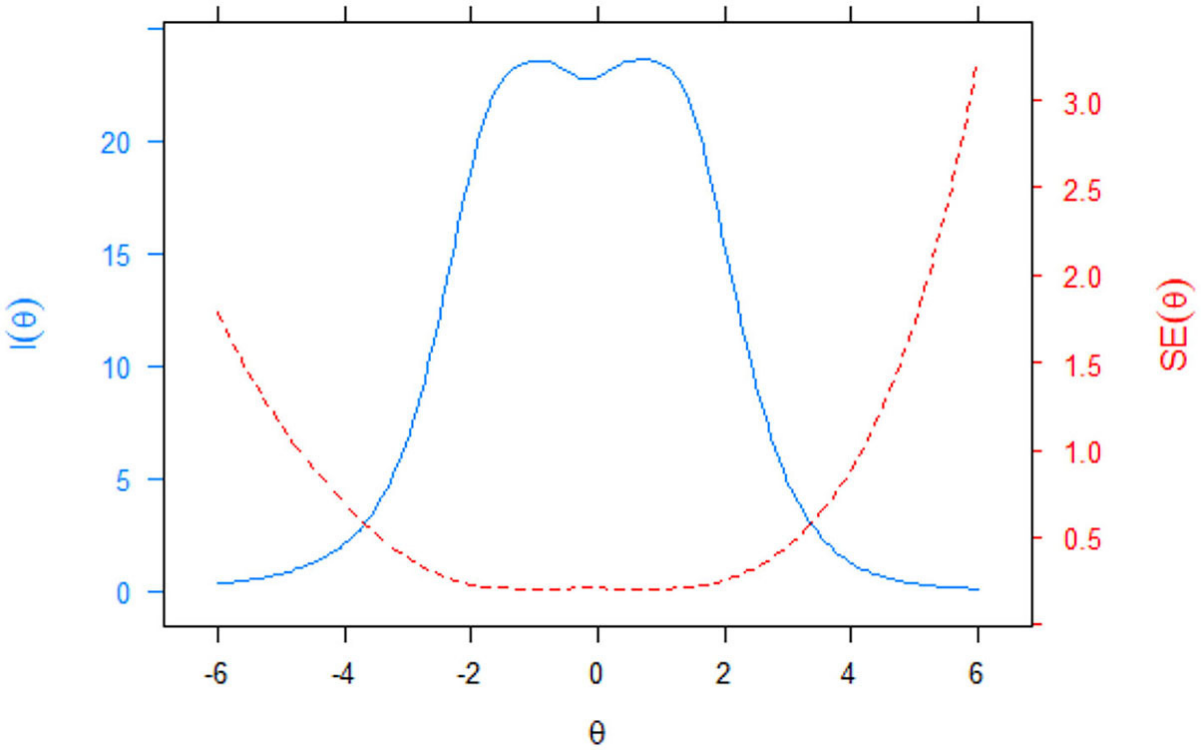
Test information and standard errors.

In this study, the marginal reliability of the long CEIS is 0.933, while the short version's marginal reliability was 0.799. Both of them met the requirement of psychometrics. Compared with the original scale, the short version saves more than 75% of the time, effectively reducing samples' cognitive fatigue and improving efficiency. Test information is 22.443 (see Figure 3).

## Pearson correlation between short and original CEIS

Expect a posteriori (EAP) was used to calculate the sample's theta. Firstly, we separately estimated the theta of the long and short versions. The former is the original scale which has 28 items (CEIS-28). The latter is the new version with 6 items (CEIS-6). Using IRT, the Pearson correlation of person parameter (theta) between the long (CEIS-28) and short (CEIS-6) version is 0.922. We can find that they have a high correlation. Furthermore, the effect of the short scale was similar to that of the long scale. Therefore, the short scale can be used instead of the long scale in a large sample study, especially when the time is limited. Then, this study calculated the total score of the short CEIS and got a Pearson correlation between CEIS-6 and each item. Items host a radio program, create a piece of artistic and functional furniture, play a musical instrument, sketch a picture, perform on stage for a group of people, and write a novel. Pearson correlation between CEIS-6 total score and its every item is 0.718, 0.656, 0.694, 0.672, 0.703, 0.604 and *p* < 0.01. These show that this study develops a short CEIS among Chinese freshmen that meet the statistical measurement requirements.

## Discussion

In order to reduce the cognitive load of respondents and improve the quality of results, this study develops short CEIS among Chinese freshmen. All the items came from Su's developed vocational interest scale, which came from the existing scale (Su et al., 2019b). Creative Expression Interest consists of seven sub-dimensions. Under the framework of IRT, after the unidimensionality test, monotonicity test, local independence test, item parameter estimation, and gender equivalence test, researchers developed a short CEIS with 6 items. These six items belong to one of the six sub-dimensions, consistent with the Single-item measures hypothesis (Postmes et al., 2013; Zhang and Wei, 2019). The seventh dimension is related to food processing and catering: select ingredients to prepare food, create the recipe for a new dish, create a new cooking technique to enhance flavor, and learn about the required temperature and time for baking pastries. In Chinese culture, the traditional view is that food processing and catering should belong to the service industry. Few people would associate this with art. In fact, with the change in the central contradiction in our society, people are seeking a better quality of life. Citizens may pay more attention to the beauty of appearance. So, the researchers need to pay more attention to this aspect in future studies.

The IRT analyses of unidimensionality, local independence, item fit, discrimination, and DIF were sequentially performed until all remaining items of the expressive art interest scale sufficiently satisfied the above rules (unidimensionality, local independence, good item-fit, high discrimination, and no DIF). Items that satisfied all the following criteria are included in the short CEIS: measuring at least one expressive art interest aspect, satisfying the hypothesis of measuring one primary dimension in IRT, satisfying the hypothesis of local independence in IRT, fitting the IRT model well, having high discrimination, and having no DIF. Subsequently, using the optimal model based on the test-level model-fit check, the item, and theta parameters of the final items were re-estimated for the IRT *via* software R4.1.1, such as Mirt, Psych, Mokken, and so on the package.

The results show that Chinese freshmen' CEIS contains six items, the specific content as follows: host a radio program, create a piece of artistic and functional furniture, play a musical instrument, sketch a picture, perform on stage for a group of people, and write a novel.

## Limitation and future research

We eliminated redundant, uninformative items from the CEIS-28 to develop a revised CEIS-6. The reduced length of the CEIS version could improve the future efficient measurement of creative expression interest. At the same time, it could also be used as a vocational assessment tool in the art field. Although this study develops a short CEIS among Chinese freshmen, our study still has some limitations:

- The researchers adopted a convenient snowball method to recruit samples. As a result, the number of women was much more than men. Therefore, we should pay more attention to the balance of subjects in future studies.
- The sample only contains Chinese freshmen, which is not conducive to promoting the results. Future research should consider the role of cultural differences and conduct cross-cultural research to test the scale of this study.
- This study only develops a short CEIS. The validity needs further testing in future research.

## Data availability statement

The raw data supporting the conclusions of this article will be made available by the authors, without undue reservation.

## Ethics statement

The study involved human participants and was reviewed and approved by the morality and ethics committee of the School of Psychology, Guizhou Normal University. The participants provided oral informed consent to participate in this study. Before conducting the questionnaire, each participant was informed of the individual privacy protection principle, and then they volunteered to participate in the survey.

## Author contributions

PZ is responsible for data analysis, method selection, manuscript writing, and revision. XG is responsible for manuscript selection, data integration, and analysis. NZ is responsible for data analysis. ZL is responsible for the manuscript communication with the finalization of the manuscript. All authors contributed to the article and approved the submitted version.

## Funding

This work was supported by the National Social Science Foundation of China General Topics in Education-Localized Research and Application of Value-added Evaluation Models (BGA210060).

## Conflict of interest

The authors declare that the research was conducted in the absence of any commercial or financial relationships that could be construed as a potential conflict of interest.

## Publisher's note

All claims expressed in this article are solely those of the authors and do not necessarily represent those of their affiliated organizations, or those of the publisher, the editors and the reviewers. Any product that may be evaluated in this article, or claim that may be made by its manufacturer, is not guaranteed or endorsed by the publisher.
